# Bibliometric visualization of global research trends in meningococcal meningitis

**DOI:** 10.1097/JS9.0000000000002920

**Published:** 2025-07-07

**Authors:** Yan Zhou, Min Liu, Rongyan Yang, Xiaobing Jiang

**Affiliations:** a Department of Neurosurgery, Union Hospital, Tongji Medical College, Huazhong University of Science and Technology, Wuhan, China; b Department of Endocrinology, The Central Hospital of Wuhan, Tongji Medical College, Huazhong University of Science and Technology, Wuhan, China; c Key Laboratory of Pollution Processes and Environmental Criteria of Ministry of Education, Tianjin Key Laboratory of Environmental Remediation and Pollution Control, College of Environmental Science and Engineering of Nankai University, Tianjin, China

## Background

In recent years, emerging infectious diseases, such as COVID-19 and monkeypox, have severely impacted the global healthcare system[1]. Meningococcal meningitis, caused by *Neisseria meningitidis*, remains a significant global public health threat despite being first identified over a century ago[2]. It progresses rapidly and has high morbidity and mortality rates. About 10% of cases are fatal, particularly among children and young adults. Additionally, about 20% of survivors suffer long-term disabilities^[3,4]^. Despite the recent advancements in vaccine technology, challenges persist due to emerging serogroup dynamics, antimicrobial resistance, and epidemic potential^[5,6]^. Therefore, understanding its epidemiology, clinical features, and research trends is crucial for public health strategies and vaccination programs.

Bibliometric analysis, a quantitative research method, is a valuable tool for mapping research patterns, identifying gaps in existing research, and highlighting research focus in specific fields^[7,8]^. It has been widely used in medicine and health; however, a bibliometric study focusing specifically on meningococcal meningitis is lacking.

## Methods

In this study, the Web of Science Core Collection (WoSCC) was searched on 5 April 2025, using terms “meningococcal meningitis” or “epidemic cerebrospinal meningitis” in titles, abstracts, and keywords. Our analysis covered English-language publications from 1997 to 2025, including original articles and reviews. After removing duplicates, the final dataset included 658 entries (582 articles and 76 reviews). The TITAN Guideline 2025 was used to transparently report the use of AI in this study[9].

**HIGHLIGHTS**

- This study provided the first bibliometric analysis of meningococcal meningitis.
- Main research themes in meningococcal meningitis were identified, along with notable countries, institutions and authors.
- This analysis provided valuable insights into the prevailing hotspots, emerging trends and gaps in the research, guiding the direction of future investigations.

## Results

**Figure 1.**
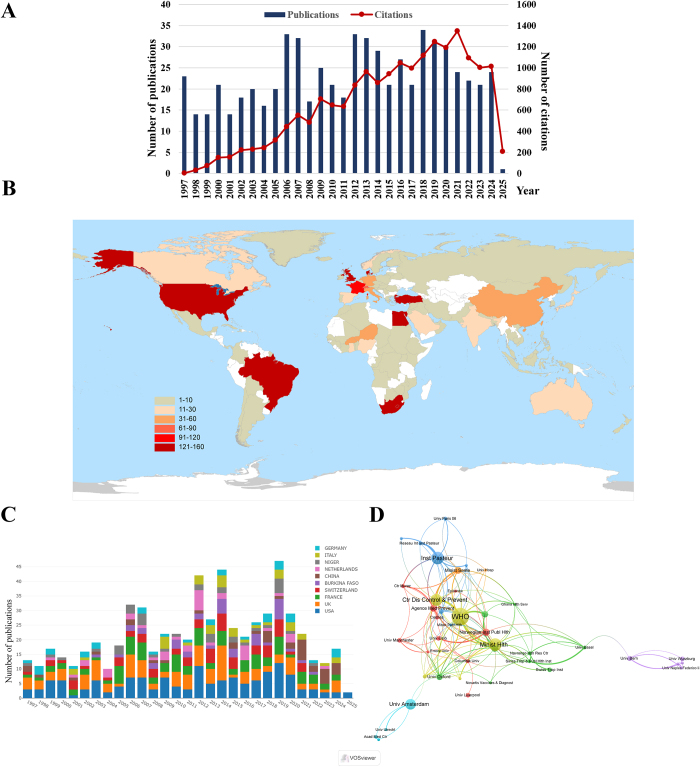
**Publication trends of articles related to meningococcal meningitis from 1997 to 2025.** (A) Number of annual publications and total citations. (B) World map displaying the number of articles per country/region, with color coding representing publication counts. (C) Annual publication trends from the top 10 contributing countries/regions. (D) Institution collaboration map generated using VOSviewer. The node size corresponds to publication counts, and the line thickness reflects collaboration strength.

The research trend on meningococcal meningitis is illustrated in Figure 1A. The number of publications gradually increased from the late 1990s, with a notable increase starting around the year 2000. This upward trend continued with some fluctuations, reaching its peak in the early 2010s. During the COVID-19 pandemic, the number of publications notably decreased, suggesting a shift in public health priorities in the pandemic. Concurrently, the pandemic delayed meningitis vaccinations for over 50 million African children, escalating the risk of a group A meningitis outbreak[10].

A total of 102 countries/regions have contributed to the meningococcal meningitis research (Fig. 1B). The USA and the UK have been the principal contributors, with the USA leading with 153 publications (23.3% of the total), followed by the UK with 120 publications (18.2%). Notably, among all African countries, Burkina Faso and Niger, situated in sub-Saharan Africa’s “meningitis belt”, contributed the most to this domain (Fig. 1B,C). Regarding specific institutions, the World Health Organization (WHO) contributed the largest number of publications (n = 50). The collaboration network of institutions is shown in Figure 1D.

**Figure 2.**
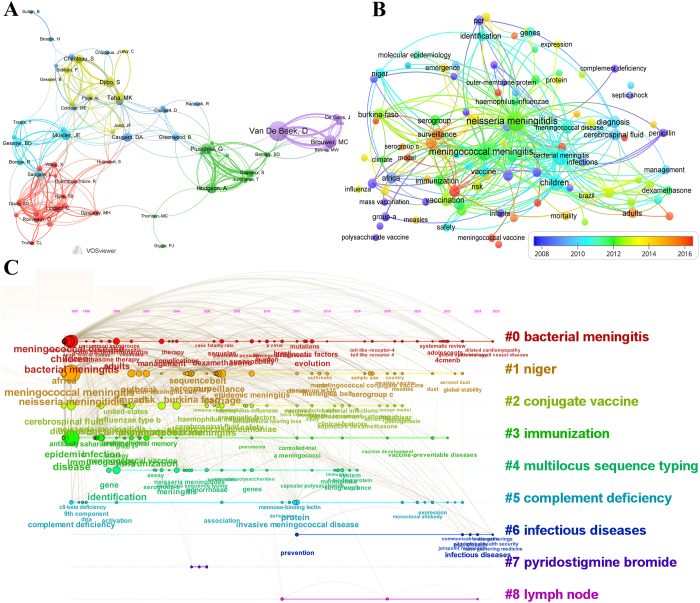
**Author collaboration and keyword analysis in meningococcal meningitis research.** (A) Author collaboration map generated using VOSviewer. The node size corresponds to publication counts, and the line thickness reflects collaboration strength. (B) Overlay visualization map of co-occurring keywords, color-coded by their average occurrence time, highlighting the temporal trends in research focus. (C) Timeline view of co-occurring keywords created using CiteSpace, showing the evolution of research themes over time. Nine labeled clusters are color-coded on the right, with nodes on the line representing co-occurring keywords.

Out of 3,303 authors, Diederik van de Beek from the University of Amsterdam has made the most significant contribution, with a total of 25 publications. Dominique A. Caugant from the Norwegian Institute of Public Health and Matthijs C. Brouwer from the University of Amsterdam have made the 2nd and 3rd most significant contributions with 16 and 15 publications, respectively (Fig. 2A). The analysis of co-authorship was illustrated using a network visualization map. The map revealed the formation of six distinct research clusters, each represented by a unique color and anchored by one or two key researchers.

In the keyword co-occurrence analysis map, node size indicates keyword frequency, and colors reflect their average emergence year (Fig. 2B). Prominent terms, such as “*Neisseria meningitidis*”, “meningococcal meningitis”, “children”, “vaccination”, “carriage”, and “diagnosis” were key research focuses. The recently emerged keywords are marked in red, highlighting trending areas. Overall, the disease impacts society and public health, surveillance, prevention, vaccine development, administration, and prognostic factors are the widely studied trending research areas. In order to track the evolution of research hotspots, the timeline of co-occurring keywords was analyzed (Fig. 2C). The visualization indicated that bacterial meningitis (#0), niger (#1), conjugate vaccine (#2), immunization (#3), multi-locus sequence typing (#4), complement deficiency (#5), infectious diseases (#6), pyridostigmine bromide (#7) and outcome (#15) were the most prominent research topics.

## Discussion

*Neisseria meningitidis* vaccination plays a crucial role in controlling epidemics and reducing disease burden. However, the current immunization practices face challenges, such as inconsistent vaccination schedules and varying initial vaccination ages[6]. To address these issues, further research is needed for vaccine-preventable bacterial diseases, such as meningococcal infections. Enhanced surveillance and diagnostic capacity might provide critical epidemiological evidence to inform the development and optimization of local vaccination policy.

This study has several limitations. First, this study only utilized the WoSCC database. Relevant studies in other databases, such as Scopus and PubMed, might have been overlooked. Second, English-language bias and the exclusion of gray literature might have missed some important research studies.

## Summary

This study presented the first comprehensive bibliometric analysis of meningococcal meningitis. Through the concerted efforts by African nations and other countries worldwide, the incidence of meningococcal meningitis has gradually declined over the years. However, more than 400 million Africans remain at risk of the seasonal meningitis epidemic, a disease that has been neglected for too long. This study underscored the critical need for sustained attention and proactive measures to effectively address this persistent threat.

## Data Availability

All data presented in this study are included in this article.
